# New liquid supports in the development of integrated platforms for the reuse of oxidative enzymes and polydopamine production

**DOI:** 10.3389/fbioe.2022.1037322

**Published:** 2022-11-28

**Authors:** Flávia F. Magalhães, Ana F. Pereira, Mara G. Freire, Ana P. M. Tavares

**Affiliations:** Department of Chemistry, CICECO-Aveiro Institute of Materials, University of Aveiro, Aveiro, Portugal

**Keywords:** laccase, polymerization of dopamine, polydopamine, aqueous biphasic systems, ionic liquids, integrated process

## Abstract

Polydopamine (PDA), a bioinspired polymer from mussel adhesive proteins, has attracted impressive attention as a novel coating for (nano) materials with an adequate conformal layer and adjustable thickness. Currently, PDA is obtained from dopamine chemical oxidation under alkaline conditions, limiting its use in materials sensible to alkaline environments. Envisaging a widespread use of PDA, the polymerization of dopamine by enzymatic catalysis allows the dopamine polymerization in a large range of pHs, overcoming thus the limitations of conventional chemical oxidation. Moreover, the conventional method of polymerization is a time-consuming process and produces PDA films with poor stability, which restricts its applications. On the other hand, the main bottleneck of enzyme-based biocatalytic processes is the high cost of the single use of the enzyme. In this work, laccase was used to catalyse dopamine polymerization. To improve its performance, a liquid support for integrating the laccase and its reuse together with the PDA production and recovery was developed using aqueous biphasic systems (ABS). Firstly, dopamine polymerization by laccase was optimized in terms of pH, temperature and initial dopamine concentration. It was demonstrated that the highest enzymatic polymerization of dopamine was achieved at pH 5.5, 30°C and 2 mg ml^−1^ of dopamine. Then, ABS composed of polymers, salts and ionic liquids were evaluated to optimize the laccase confinement in one phase while PDA is recovered in the opposite phase. The most promising ABS allowing the separation of laccase from the reaction product is composed of polypropylene glycol (400 g mol^−1^) and K_2_HPO_4_. The polymerization of dopamine in ABS leads to a remarkable improvement of polymerization of 3.9-fold in comparison to the conventional chemical PDA polymerization. The phase containing the confined laccase was reused for four consecutive reaction cycles, with a relative polymerization of 68.9% in the last cycle. The results of this work proved that ABS are a promising approach to create a liquid support for enzyme reuse allowing the process intensification efforts. The use of biocatalysts in ABS emerges as sustainable and alternative platforms from environmental and techno-economic points of view.

## 1 Introduction

Polydopamine (PDA) is a bioinspired polymer from mussel adhesive proteins, and has attracted relevant attention as a novel coating for (nano) materials with adequate conformational layer and adjustable thickness (Lee et al., 2007). PDA was first introduced in 2007 by Lee et al. (2007) as a multifunctional coating for different materials including noble metals, oxides, ceramics and polymers (Cai et al., 2018; Song et al., 2021; Teng et al., 2021). Since then, PDA has been widely used in the modification and functionalization of several (nano) materials, providing them new functionalities for a diversity of applications, especially in the field of biomedicine (Cai et al., 2018; Tripathi et al., 2018; Wu et al., 2022). Currently, PDA can be used as surface modifier for tissue engineering, cell adhesion, capsules, biosensing and drug delivery (Ding et al., 2016; Ball, 2018; Singh et al., 2021). The outstanding adhesive properties of PDA are attributed to the presence of catechol, amino and imine functional groups which can bind by covalent bonds or physical adsorption (Yan et al., 2020). Moreover, the (nano) materials modified with PDA present biocompatibility, biodegradability and antioxidative properties (Zhou et al., 2020). Comparing to other coating methodologies, such as chemical vapor deposition and layer-by-layer deposition (complex, expensive equipment, multiple steps and only applicable to certain materials) PDA coating can be achieved in a single-step (easy processing) allowing the functionalization of a large number of surfaces (El Yakhlifi and Ball, 2020).

The conventional method for dopamine polymerization is under alkaline conditions (pH > 8.5) that leads to spontaneously dopamine self-polymerization. However, it is a time-consuming process requiring a long reaction time, 24 h, and oxidant agents such as O_2_, NaIO_4_ or KClO_3_ (Wei et al., 2010; Li et al., 2018). These hard conditions have a lot of drawbacks when coating biological, alkali-sensitive or other surfaces that are not compatible with the reaction conditions such as polyester, phenolic resin and proteins, thus, limiting the widespread applications of PDA coatings (Li et al., 2018). In addition, the use of hazardous chemicals produces large quantities of insoluble precipitates, besides leading to rough surfaces and difficulty in controlling PDA film thickness (Lee et al., 2007). To overcome these limitations, a biocompatible, efficient, and environmentally friendly approach based on biocatalysis with oxidative enzymes has been recently proposed for the polymerization of dopamine since a well-controlled and material-efficient thin film formation can be produced (Kobayashi and Makino, 2009; Milyaeva et al., 2020). Laccases (oxygen oxidoreductase, EC 1.10.3.2) are multicopper oxidases with high catalytic efficiency for the degradation of both phenolic and non-phenolic compounds and for the synthesis of polymers (Walde et al., 2019; Mayolo-Deloisa et al., 2020). Moreover, due to their broad substrate range and diversity of biotechnological applications they are widely employed in the industrial sector (Zdarta et al., 2018; Mayolo-Deloisa et al., 2020; Othman et al., 2022). Therefore, because of the diphenolic structure of dopamine, laccases are a potential biocatalyst for its oxidative polymerization in a large range of pH values (Olmeda et al., 2021). For example, Li et al. (2018) compared the enzymatic polymerization of dopamine using free laccase from *Trametes versicolor* with the conventional method. According to this study, the enzymatic polymerization of dopamine at pH 5.5 was ∼3.4-fold higher and the obtained PDA films were more uniform and stable than the films produced by the conventional method (pH 8.5 under aerobic conditions) (Li et al., 2018). However, the use of free laccase has some limitations such as the loss of activity and stability as well as difficulties in its recovery and reusability increasing the process costs (Ferreira et al., 2018; Zhang et al., 2020; Adamian et al., 2021). To boost the economic and sustainable viability of the enzymatic process for dopamine polymerization with laccase, the reuse of the enzyme without loss in its biological properties is an ever-increasing demand for industrial applications (Zdarta et al., 2018). In this context, the most used approach is the enzyme immobilization on a solid support (Gan et al., 2021). Nevertheless, this strategy might lead to activity loss and conformational changes in the enzyme structure (Shakerian et al., 2020). Therefore, there is an urgent need to find alternative techniques to overcome such problems and ensure the biocatalyst’s recovery and reusability from the reaction medium. When appropriately designed, aqueous biphasic systems (ABS), which are composed of water and at least two water-soluble components [e.g., polymers, salts, ionic liquids (ILs)], appear as an excellent and promising alternative with outstanding environmental and economic points of view. ABS consist of two immiscible aqueous-rich phases formed when water-soluble components are mixed above given concentrations (Freire et al., 2012; Pereira et al., 2020). Recently, ABS systems have been investigated as reaction media and liquid supports for enzymes since they provide a suitable and friendly environment for the maintenance of the enzymatic activity and its reuse (Ferreira et al., 2018; Capela et al., 2020; Ferreira et al., 2021; Muñiz-Mouro et al., 2021). Another advantage and application of this approach is the possibility of simultaneous extract and concentrate the reaction product by manipulating the volume of the ABS phases, using the tie-line of binodal curves (Dinis et al., 2018), while maintaining the enzyme in the opposite phase. Besides, the process integration is the principal strength of this technique: enzymatic reactions can be integrated with the separation step, thus, making an integrated biocatalytic process (Pereira et al., 2020; Magalhães et al., 2021). Thereby, it is possible the separation of the target product and the reuse of the enzyme in a unique step, contributing to a reduction in process costs and time. In a recent study assessed by. Ferreira et al. (2021) it was demonstrated the recovery and reuse of laccase in an integrated reaction-separation process by applying thermoreversible ABS. No losses in the enzymatic activity were observed for at least five consecutive cycles of enzymatic reaction with 2,2′-azino-bis (3-ethylbenzothiazoline-6-sulphonic acid) (ABTS). In the work performed by Muñiz-Mouro et al. (2021), the oligomerization of rutin using laccase as the biocatalyst was carried out in ABS. It was verified a preferential migration of laccase to the opposite phase of the product throughout the cycles and its reuse in three reaction-separation cycles was achieved.

This work aims the development of an integrated and sustainable platform for the enzymatic production and recovery of PDA and biocatalyst reuse using ABS. The biocatalytic process for the polymerization of dopamine was carried out using laccase as the biocatalyst and ABS composed of polymers, salts, and ILs. ABS was used for the first time as reaction media and liquid support for laccase allowing the simultaneous separation of product, soluble PDA intermediate species (PDA_i_), and the reuse of the enzyme. The potential of laccase in polymerization is herein exploited and compared with the non-enzymatic method. Several parameters including the temperature, pH of the medium and initial dopamine concentrations on the dopamine polymerization were also investigated and optimized.

## 2 Materials and methods

### 2.1 Materials

Polypropylene glycol with a molecular weight of 400 g mol^−1^ (PPG 400), polyethylene glycol with a molecular weight of 400 g mol^−1^ (PEG 400), ABTS (≥ 98 wt% purity) and dopamine hydrochloride (98 wt% purity) were acquired from Sigma-Aldrich. The ILs, cholinium dihydrogen phosphate ([Ch][DHP], >98 wt% purity) and cholinium acetate ([Ch][Acet], 98 wt% purity) were both purchased from IoLiTec, and cholinium dihydrogen citrate ([Ch][DHC], ≥98% wt% purity) from Sigma-Aldrich. Dipotassium hydrogen phosphate trihydrate (K_2_HPO_4_.3H_2_O, 98 wt% purity) was purchased from Scharlau.

Commercial laccase from *Trametes versicolor* (10 U mg^−1^) was acquired from Sigma-Aldrich. Sodium acetate (CH_3_COONa) pure from AnalaR Normapur and acetic acid (CH_3_COOH, 99 wt% purity) from Fisher Chemical was used to prepare sodium acetate buffer; disodium phosphate (Na_2_HPO_4_, 99 wt% purity) and citric acid (C₆H₈O₇, 99.5 wt% purity), both acquired from Panreac were used to prepare citrate-phosphate buffer; Tris-HCl buffer was prepared by using tris (hydroxymethyl) aminomethane [NH_2_C(CH_2_OH)_3_, >99 wt% purity] from PRONALAB and a hydrochloric acid solution (HCl, 1 M).

### 2.2 Optimization of the enzymatic polymerization of dopamine

Enzymatic polymerization of dopamine was performed according to the adapted procedure by Li et al. (2018). To optimise the dopamine polymerization into PDA_i_ using laccase, several reactional parameters were studied, namely pH, temperature, and initial dopamine concentrations.

The pH effect on enzymatic polymerization was studied using sodium acetate buffer for pHs 4.5 and 5.5; phosphate buffer for pH 6.5 and Tris-HCl buffer for pH 7.5 and 8.5 (0.05 M). Dopamine solution (2.00 mg ml^−1^) was prepared in the multiple buffers and laccase (1.00 mg ml^−1^) was added to perform the enzymatic reaction. The reactions were kept at a controlled temperature (30°C) and continuously magnetic stirred (350 rpm) at atmospheric air for 1 h.

The influence of temperature was investigated. The assay was performed by adding a dopamine solution (2.00 mg ml^−1^) prepared in sodium acetate buffer at pH 5.5 (0.05 M) and laccase (1.00 mg ml^−1^). The reactions were performed at a controlled temperature in a range from 20 to 40°C and continuously magnetic stirred (350 rpm) at atmospheric air for 1 h.

The effect of dopamine concentration was evaluated by adding a dopamine solution with different concentrations ranging from 0.25 to 3.00 mg ml^−1^ prepared in sodium acetate buffer at pH 5.5 (0.05 M) and laccase (1.00 mg ml^−1^). The reactions were kept at a controlled temperature (30°C) and continuously magnetic stirred (350 rpm) at atmospheric air for 1 h. Each assay was performed in triplicate. Reactions without laccase under the same conditions were carried out and considered as control.

UV−vis spectroscopy was employed to monitor the oxidation of dopamine. The degree of dopamine polymerization was indirectly monitored at 305 and 480 nm using a UV-Vis spectrophotometer (Shimadzu UV-1800 Spectrometer). Both wavelengths correspond to PDA_i_. For this purpose, spectra were acquired in wavelength range from 250 to 500 nm.

The relative enzymatic polymerization of dopamine in relation to the non-enzymatic was determined according to Eq. 1. The absorbance of the non-enzymatic polymerization was obtained after 1 h of reaction under the best conditions previously optimised (35°C, pH 8.5 and dopamine 2.00 mg ml^−1^) and was used as a reference value in the calculation of the relative enzymatic polymerization.

$$Relative\;Polymerization\;\left(fold\right)=\frac{abs\;\left(enzymatic\;\right)}{abs\;\left(non-enzymatic\right)}$$ (1)

where abs (enzymatic) and abs (non-enzymatic) correspond to the absorbance values at 480 nm of the laccase-catalysed polymerization and non-enzymatic polymerization, respectively.

### 2.3 Laccase activity method

The laccase activity was measured by adding 50 μl of sample to 250 μl of ABTS aqueous solution and 700 μl of citrate phosphate buffer (0.1 M, pH 4.5). The increase in absorbance per min was spectrophotometrically measured at 420 nm and expressed in U·L^−1^. Each measurement was analysed in triplicate. 1 Unit (U) of laccase activity corresponds to the amount of enzyme necessary for the oxidation of 1 μmol of the substrate (ABTS) per min (*ε* = 36.000 M^−1^ cm^−1^).

#### 2.3.1 Effect of dopamine and PDAi in the laccase activity method

The enzymatic activity of laccase was evaluated in the presence of dopamine by adding 200 μl of dopamine solution with different concentrations ranging from 0.10 to 1.00 mg ml^−1^ [prepared in Tris-HCl buffer at pH 8.5 (0.05 M)] and 200 μl of laccase (0.60 mg ml^−1^) + 600 μl Tris-HCl buffer. Then, laccase activity was measured as previously described. The laccase activity measured in Tris-HCl buffer pH 8.5 was considered as the control (100%).

To evaluate the influence of PDA_i_ in the laccase activity method, the polymerization of dopamine was carried out by the traditional method using Tris-HCl buffer at pH 8.5 (0.05 M). 200 μl of PDAi solution was added to 200 μl of laccase solution (0.60 mg ml^−1^) + 600 μl Tris-HCl. Then, laccase activity was measured as previously described. The laccase activity in the Tris-HCL buffer pH 8.5, used in the production of PDA_i_, was considered as the control (100%).

### 2.4 Evaluation of the extraction efficiency of laccase, dopamine and polydopamine in ABS

The extraction efficiency of active laccase (*EE*_*Laccase*_%), dopamine (*EE*_*dopamine*_%) and PDA_i_ (*EE*_*PDA*_%) was evaluated in five different ABS. The ABS ternary mixture compositions used were based on the previous binodal curves reported in the literature (Pereira et al., 2015; Sadeghi and Maali, 2016): 45 wt% PPG 400 + 7 wt% [Ch][DHP] + 48 wt% H_2_O; 46 wt% PPG 400 + 16 wt% [Ch][DHC] + 38 wt% H_2_O; 51 wt% PPG 400 + 6 wt% [Ch][Acet] + 43 wt% H_2_O; 33 wt% PPG 400 + 6.4 wt% K₂HPO₄ + 60.6 wt% H_2_O and 46 wt% PPG 400 + 24 wt% PEG 400 + 30 wt% H_2_O.

*EE*_*Laccase*_% was determined by adding 10 wt% of an aqueous laccase solution (1.00 mg ml^−1^) to the ABS mixture compositions described above, replacing the water content, to attain a total ABS weight of 2 g. After the homogenization of the ABS, the phase separation was carried out by centrifugation at 3,500 rpm for 20 min at 25°C. The *EE*_*Laccase*_ % was considered as the percentage ratio between the laccase activity in the bottom phase (bot) to that in the opposite top phase (top), according to Eq. 2. For each experiment three replicates were prepared.

$$EE_{Laccase}\;\%=\frac{Laccase\;activity\;\left(bot\right)*W\left(bot\right)}{Laccase\;activity\;\left(bot\right)\;*\;W\left(bot\right)+Laccase\;activity\;\left(top\right)\;*W\left(top\right)}\times \mathrm{100}$$ (2)

where W (bot) and W (top) correspond to the weight of the bottom and top phases, respectively.

To evaluate *EE*_*dopamine*_ *%* for each ABS mentioned, 20 wt% of an aqueous dopamine solution (2.00 mg ml^−1^) was added to the ABS mixture compositions described above, replacing the water content, attaining a total ABS weight of 2 g. After the homogenization of the ABS, the phase separation was carried out by centrifugation at 3,500 rpm for 20 min at 25°C. The absorbance of each phase was measured at 280 nm using a Shimadzu UV-Vis spectrometer. *EE*_*dopamine*_ *%* was determined according to Eq. 3. For each experiment three replicates were prepared.

To evaluate *EE*_*PDA*_ %, produced by the enzymatic catalysis, the polymerization of dopamine was carried out using the previously mentioned five ABS at the same compositions. For each ABS, 20 wt% of dopamine solution (2.0 mg ml^−1^) and 10 wt% laccase aqueous solution (1.0 mg ml^−1^) were added to the ABS mixture compositions, replacing water content, for total ABS weight of 2 g. The reaction was incubated at 25°C and stirred for 1 h. Afterwards, both phases of the ABS were separated after centrifugation at 3,500 rpm for 30 min at 25°C. PDA_i_ partition was assessed by recording the UV-Vis absorbance spectra ranging between 250 and 500 nm, through the analysis of absorbance at 480 nm. *EE*_*PDA*_ *%* was determined according to Eq. 3. For each experiment three replicates were prepared.

$$EE_{dopamine/PDA}\;\%=\frac{abs\;\left(top\right)*\;DF\left(top\right)*\;W\left(top\right)}{abs\;\left(top\right)*\;DF\left(top\right)*\;W\left(top\right)+abs\;\left(bot\right)*DF\left(bot\right)*W\left(bot\right)}\times 100$$ (3)

where abs (top) and abs (bot) correspond to the absorbance values at 280 nm (for dopamine partition) or 480 nm (for PDA_i_ partition) of the top and bottom phases, respectively. DF corresponds to the dilution factor of each phase, W (top) to the weight of the top phase and W (bot) the weight of the bottom phase.

### 2.5 Evaluation of the dopamine concentration in the enzymatic polymerization using ABS

Laccase-catalysed dopamine polymerization was carried out in the selected ABS composed of 33 wt% PPG 400 + 6.4 wt% K₂HPO₄ + 20 wt% dopamine solutions (0.50–3.50 mg ml^−1^) + 40.6 wt% laccase solution at 2.56 mg ml^−1^. The reactions were performed for 1 h, incubated at 30°C and continuously under magnetic stirring (350 rpm). After 1 h, the phase separation was carried out by centrifugation at 3,500 rpm for 30 min at 25°C. Relative polymerization (fold) was determined according to Eq. 1. Different final dopamine concentrations from 0.10 to 0.70 mg ml^−1^ were evaluated. The conventional method of polymerization (without laccase) was also carried out in the same ABS and was considered as the reference: 33 wt% PPG 400 + 6.4 wt% K₂HPO₄ + 20 wt% dopamine solution (0.50–3.50 mg ml^−1^) + 40.6 wt% H_2_O at 30°C.

#### 2.5.1 Laccase activity in presence of ABS bottom phase

To evaluate the effect of the ABS bottom phase in the laccase activity, the selected ABS composed of 33 wt% PPG 400 + 6.4 wt% K₂HPO₄ was prepared and the phase separation was carried out by centrifugation at 3,500 rpm for 30 min at 25°C. The laccase activity was determined in the bottom phase of the ABS that was used as a medium for laccase incubation. The laccase activity measured in water at the same conditions (final enzyme concentration at 0.60 mg ml^−1^ and after 1 h of incubation time at 25°C) was considered as the control (100%).

### 2.6 Laccase recovery and reuse using ABS

ABS is used to recovery and reuse of the enzyme by confining the enzyme in the bottom phase. For the study of recovery and reuse of laccase, four consecutive cycles of dopamine polymerization were performed using the optimised ABS composed of 33 wt% PPG 400 + 6.4 wt% K₂HPO₄ + 20 wt% dopamine solution (3.00 mg ml^−1^) + 40.6 wt% laccase solution (1.00 mg ml^−1^). After the first reaction cycle, the PDA_i_-enriched top phase was removed, and the same volume of a solution containing the respective top polymer-rich phase constituents (determined by the tie-line) (Ferreira et al., 2021) was added to the remaining laccase-rich bottom phase. Dopamine solution was added to reach a final concentration of 0.60 mg ml^−1^ in the ABS and a new biocatalytic cycle started. This step was repeated for four consecutive reaction cycles. After each cycle, the ABS was centrifuged at 3,500 rpm for 30 min and both phases were separated. Laccase reuse is confirmed by the increase in dopamine polymerization.

Relative polymerization (%) was defined as the ratio between the obtained absorbance at the end of each cycle with respect to that obtained in the first cycle and was calculated according to Eq. 4:

$$Relative\;polymerization\;\left(\%\right)=\frac{abs\;\left(n\;cycle\right)}{abs\;\left(1st\;cycle\right)}\times 100$$ (4)

where abs_(n cycle)_ and abs_(1st cycle)_ correspond to the absorbance values at 480 nm obtained in the ABS top phase each cycle and the first cycle of reaction, respectively.

## 3 Results and discussion

### 3.1 Optimization of the enzymatic polymerization of dopamine

**FIGURE 1:**
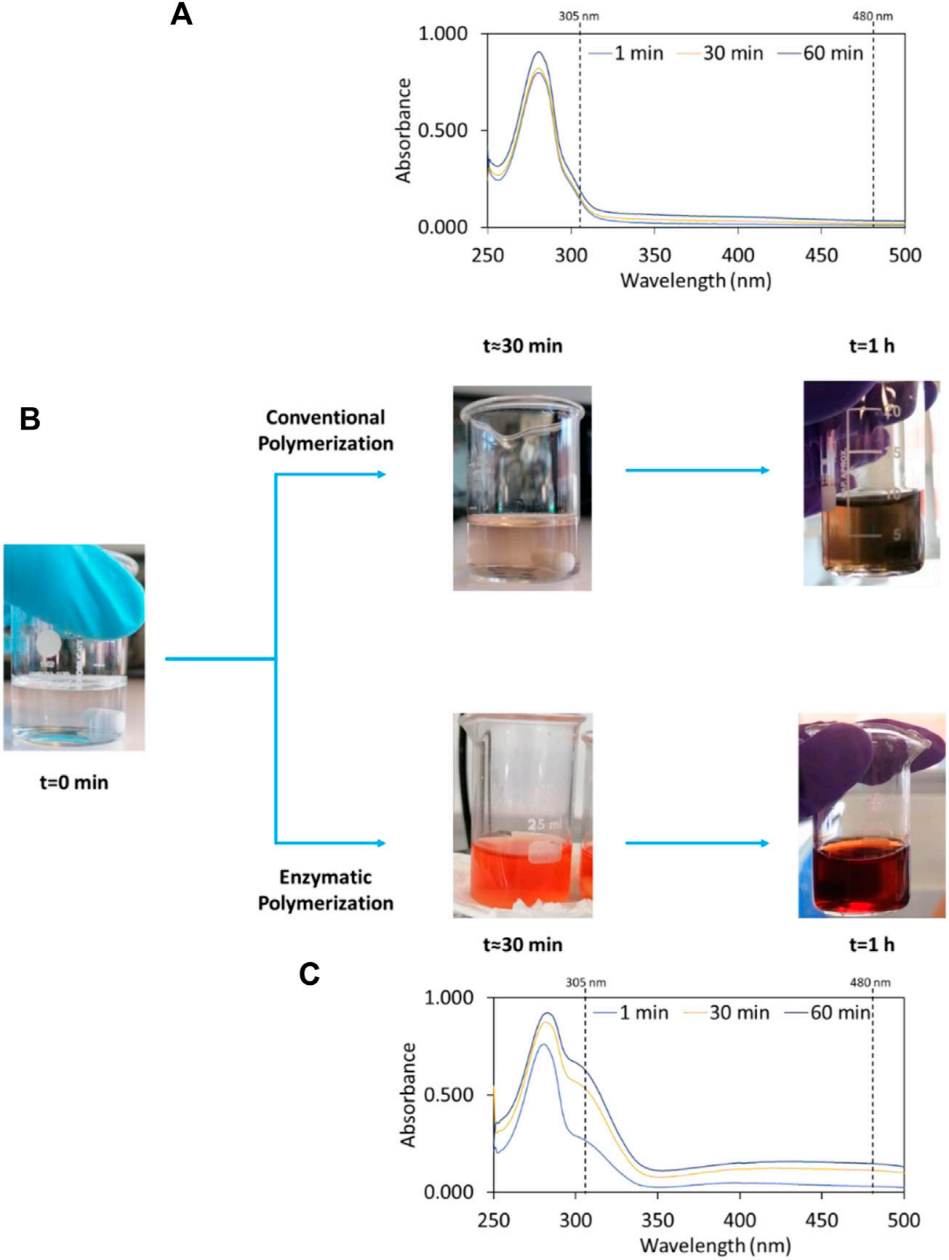
Colour changes of initial solutions of dopamine after 30 min and 1 h **(B)** and time-dependent UV-Vis absorbance spectra for: Conventional polymerization of dopamine, pH 8.5 and 30°C **(A)** and enzymatic polymerization of dopamine, pH 5.5 and 30°C **(C)**.

To explore the potential of laccase in the polymerization of dopamine, the enzymatic polymerization was investigated at pH 5.5 and compared to the alkaline conventional method at pH 8.5. Both reactions were carried out at 30°C for 1 h. In the presence of laccase, the colourless of the initial dopamine solution rapidly (∼1 min) changed to a reddish solution, indicating the fast polymerization of dopamine. After 1 h of reaction, the red colour became more intense (dark red) (Figure 1B). Comparatively, in the non-enzymatic polymerization, the colourless solution turned into pale yellow and after 1 h into grey. Small PDA films were also observed at the surface of the solution (although not so notable), as well as grains in the bulk (Figure 1B).

The oxidation of dopamine into PDA_i_ was assessed by UV-Vis absorption intensities at 305 and 480 nm, which correspond to the formation of dopamine semiquinone and dopaminechrome intermediate species, respectively. The subsequent oxidation of these species leads to the formation of PDA (Wei et al., 2010; Salomäki et al., 2018). In this study, the absorbance value chosen to evaluate and calculate the relative polymerization was 480 nm, since it corresponds to the intermediate species to produce PDA during the polymerization reaction. All UV-Vis absorbance spectra during the time of both reactions and the detailed analysis and discussion of the intermediate species are reported in the Supplementary Figures S1–S4. According to Figure 1C, there is a fast increase in absorption intensities at 305 and 480 nm of the enzymatic polymerization during the reaction time. In contrast, for the conventional non-enzymatic reaction (Figure 1A), the absorption peaks intensity remains unaltered in all wavelength ranges during the reaction time, which indicates that the polymerization of dopamine into PDA_i_ is very slow. The dopamine polymerization reaction was improved in the presence of laccase since for the same reaction time the conventional polymerization was significantly inferior. Relatively to the absorbance at 305 nm, for the enzymatic method, an absorbance increase of ∼4.6-fold in relation to the non-enzymatic method is obtained after 1 h. Regarding the absorbance at 480 nm, an increase of ∼7-fold after 1 h was obtained when compared to the non-enzymatic dopamine polymerization. These results are consistent with those reported by Li et al. (2018), in which the spectra follow the same trend at the same wavelength values.

**FIGURE 2:**
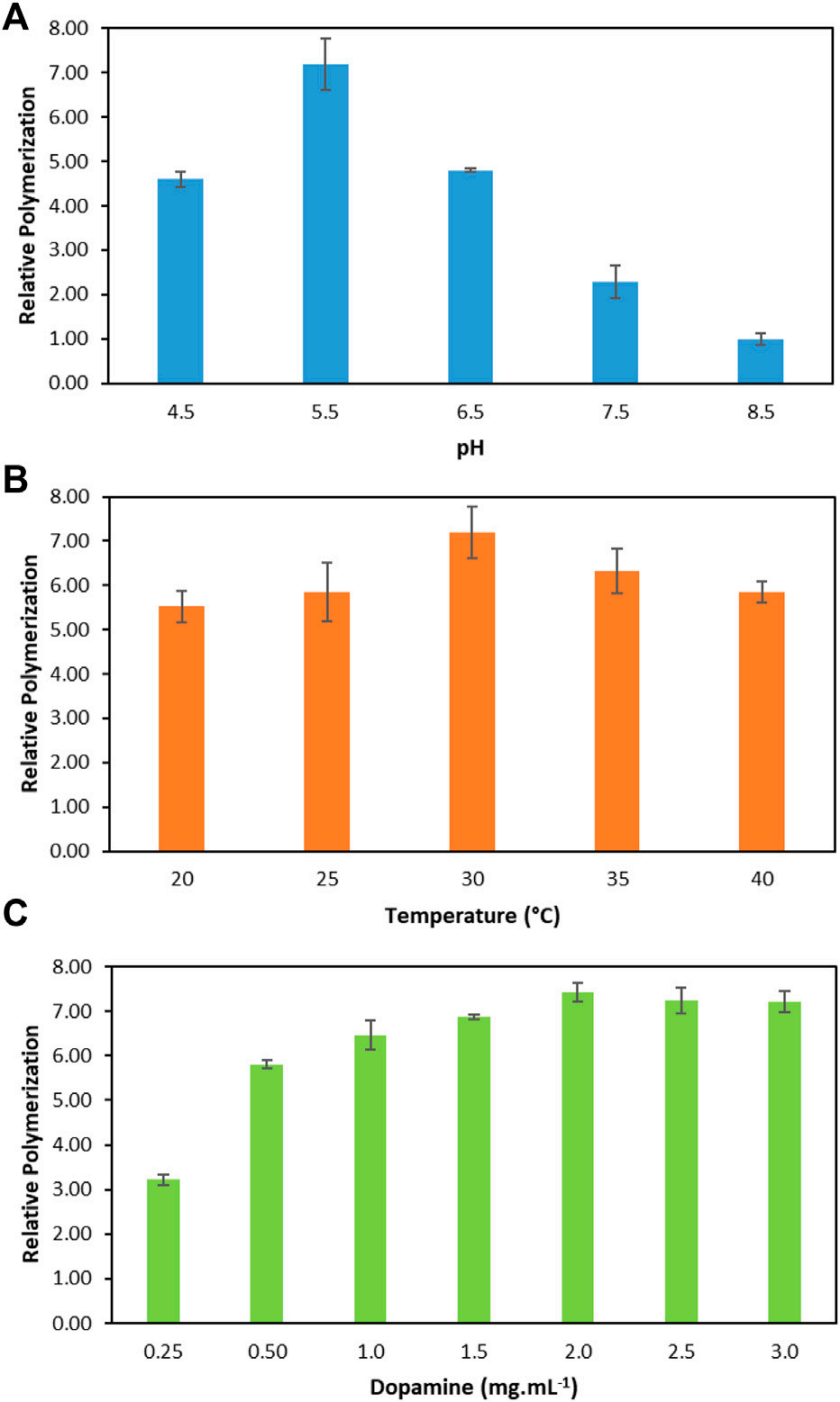
Relative dopamine polymerization in relation to the conventional reaction (without laccase, considered as 1). **(A)** At different pH values, 30°C and dopamine 2.00 mg ml^−1^. **(B)** At different temperatures, pH 5.5 and dopamine 2.00 mg ml^−1^. **(C)** At different dopamine concentrations, pH 5.5 and 30°C.

To improve the performance of the enzymatic polymerization of dopamine, the following reaction parameters were optimised: pH, temperature, and initial dopamine concentrations. Figure 2 depicts the relative increase in the polymerization of dopamine with laccase in relation to the conventional reaction (without laccase) at the best reaction condition: pH 8.5, 30°C and 2.00 mg ml^−1^. Conventional reaction was considered as the control (1-fold). It should be noted that the conventional dopamine polymerization was also evaluated at a pH range between 4.5 and 7.5 and, as expected, no polymerization was observed. The respective detailed data are reported in the Supplementary Tables S1–S3.

Firstly, the effect of the pH on the enzymatic dopamine polymerization was evaluated since the pH affects the oxidation and polymerization by laccase. The pH dependence of laccase was studied at pH values ranging from 4.5 to 8.5. The respective relative polymerization values are depicted in Figure 2A. All reactions were carried out with an initial dopamine concentration of 2.00 mg ml^−1^ at 30°C. According to the results, it is evident that the optimal pH for the enzymatic polymerization of dopamine was pH 5.5. The efficiency of the enzymatic reaction at pH 5.5 was improved by approximately 7.2-fold. In addition, all pHs ranging from 4.5 to 7.5 present better relative dopamine polymerization than the conventional polymerization. As expected, the lowest polymerization value corresponds to pH 8.5 which is similar to the conventional approach. This can be explained by the higher pH since the laccase performance decreases due to the inhibitory effect of OH^−^ on the active site of the enzyme (Tan et al., 2010). This result indicates that laccase exhibits a low capacity for dopamine polymerization in high pHs values which is in agreement with the literature (Arregui et al., 2019; Yin et al., 2019). Considering the laccase activity and relative polymerization, pH 5.5 was chosen for dopamine polymerization.

After selecting the best pH of 5.5, the temperature from 20 to 40°C was evaluated in the relative dopamine polymerization (Figure 2B). The temperature that led to the high relative dopamine polymerization was 30°C, with an increase of 7.2-fold when compared to the conventional method. In addition, an increase of 6.3-fold, 5.8-fold and 5.8-fold in the relative dopamine polymerization was obtained for 35, 40 and 25°C, respectively. These results indicate that laccase is able to polymerize dopamine in a significant temperature range. According to the literature, laccase from *T. versicolor* shows an increase in its activity with the increase in the temperature up to 60°C, however, its thermal stability stands between 10 and 30°C (Kurniawati and Nicell, 2008). The results obtained agree with these data since the best temperature to work with laccase is 30°C, where the enzyme has a good thermal stability.

At the optimum pH and temperature (pH 5.5 and 30°C), the initial dopamine concentration was optimised in the range of 0.25–3.00 mg ml^−1^. The relative dopamine polymerization values are depicted in Figure 2C. There is an increase in the relative polymerization values as the dopamine concentration increases up to 2.00 mg ml^−1^, leading to the highest relative dopamine polymerization of 7.4-fold. From 2.00 mg ml^−1^, the relative dopamine polymerization remained constant, which indicates the saturation of the enzyme by the dopamine. After the enzyme reaches its saturation point, the increase of dopamine concentration did not affect the reaction rate, creating the same amount of PDA_i_ in 1 h. Moreover, it is important to highlight that even at the lowest dopamine concentration (0.25 mg ml^−1^) a relative polymerization increase of ∼3-fold was obtained in relation to the maximum value observed for the conventional non-enzymatic method at 2.00 mg ml^−1^.

The parameters for the enzymatic dopamine polymerization were optimised (pH 5.5, 30°C and dopamine 2.00 mg ml^−1^). Under these conditions, the relative enzymatic dopamine polymerization achieves 7.4-fold in comparison to the conventional dopamine polymerization. These results confirm that the use of laccase has many advantageous for the PDAi production. In addition, the laccase reuse is important to achieve a more sustainable and economic process. Therefore, the reuse of laccase in ABS is further investigated to improve the process.

### 3.2 ABS as integrated platform for the polydopamine production and laccase reuse

#### 3.2.1 Extraction efficiency of laccase, PDA_i_ and dopamine

Enzyme reuse and substrate recovery need to be considered when developing a sustainable and economical process. The reuse of enzymes using liquid supports can provide a novel opportunity to surpass the major challenge of biocatalyst recovery and reuse while improving its stability, activity, biocompatibility and reaction rates (Ferreira et al., 2021; Muñiz-Mouro et al., 2021; Baptista et al., 2022). To this end, ABS approach was chosen as excellent liquid supports to develop an integrated and sustainable platform for enzymatic catalysis. This approach allows PDA_i_ to be recovered in one phase while the enzyme is confined into the opposite phase for further reuse.

**FIGURE 3:**
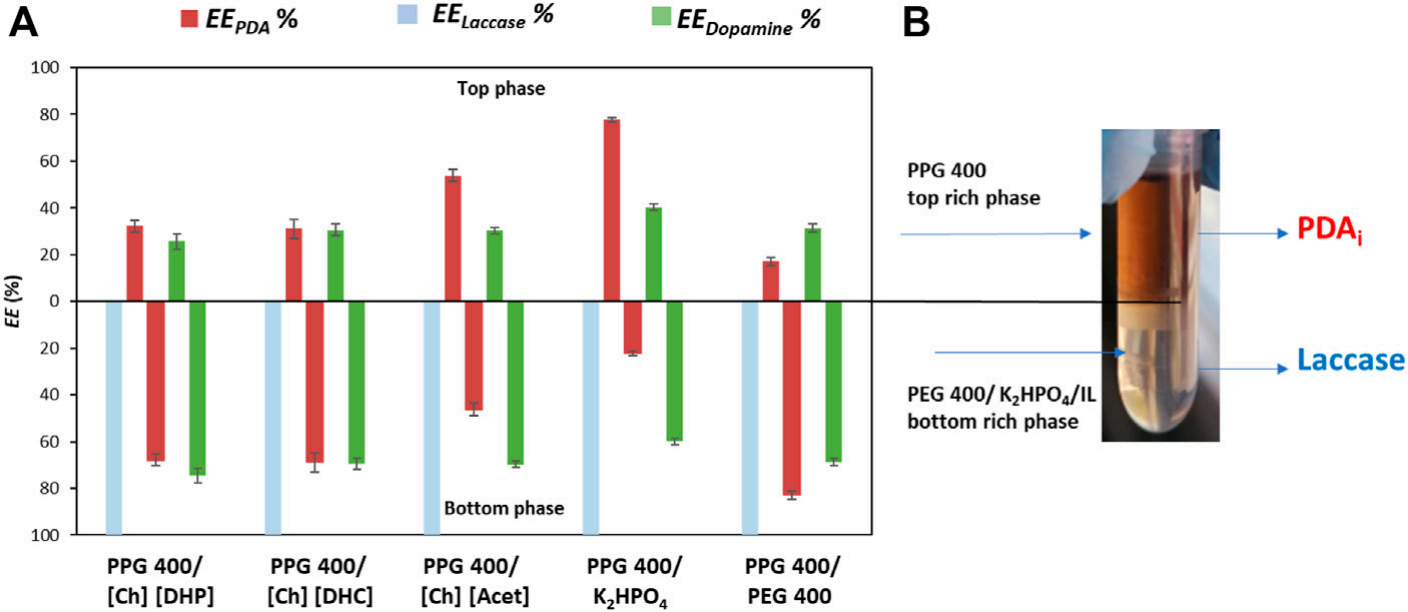
**(A)** Extraction efficiency of PDA_i_ (*EE*_*PDA*_ %, red bars), laccase (*EE*_*Laccase*_ %, blue bars) and dopamine (*EE*_*Dopamine*_ %, green bars) in the top and bottom-phase. **(B)** PPG 400 + K_2_HPO_4_ ABS after the enzymatic polymerization of dopamine and phases separation.

To develop this multifunctional platform, it is mandatory that PDA_i_ migrate to the opposite phase of laccase. Thus, it was firstly evaluated the *EE*_*PDA*_ %, *EE*_*Laccase*_ %, and *EE*_*Dopamine*_ % in five different ABS composed of PPG 400 and [Ch][DHP], [Ch][DHC], [Ch][Acet], PEG 400 or K_2_HPO_4._ The ternary mixture compositions of the ABS were selected according to the literature (Ferreira et al., 2021) and correspond to a mixture point that leads to a biphasic system with an approximately equal ratio of both phases. Thus, it is possible to ensure enough mass in each phase for laccase reaction and reuse and product recovery. The *EE*_*PDA*_ and *EE*_*Laccase*_ % are shown in Figure 3A and an image of the PPG 400 + K_2_HPO_4_ ABS after the enzymatic polymerization reaction is shown in Figure 3B. The respective detailed data are reported in the (Supplementary Tables S4–S6).

The results depicted in Figure 3A show a selective partition of laccase for the bottom phase (IL-, K_2_HPO_4_- or PEG-rich phase) of all ABS, with an *EE*_*Laccase*_ of 100%. These results are in accordance with previous works demonstrating that proteins and enzymes have a high affinity for the more hydrophilic phase (Ferreira et al., 2018; Capela et al., 2020; Ferreira et al., 2021). For example, the study carried out by Capela et al. (2020) proved that using ABS composed of cholinium-based ILs or PEG 400 + PPG 400, laccase complete partitioned for the more hydrophilic phase namely IL- or PEG 400- rich phase with an *EE*_*Laccase*_ of 100%.

Regarding the *EE*_*PDA*_ %, for the ABS composed of PPG 400 + [Ch][Acet], PDA preferentially migrates to the top phase (*EE*_*PDA*_ of 53.8%), whereas for PPG 400 + [Ch][DHP], PPG 400 + [Ch][DHC], and PPG 400 + PEG 400, PDA_i_ and laccase preferentially migrate to the bottom-phase, with an *EE*_*PDA*_ to the top-phase ranging from 17.2 to 32.2%. Thus, these ABS were excluded for further studies since the PDA_i_ was in the same phase of laccase. On the other hand, the ABS composed of PPG 400 + K_2_HPO_4_ led to the best *EE*_*PDA*_ of 77.8%. PDA_i_ preferentially migrated to the PPG-rich top phase while laccase complete migrated to the salt-rich bottom phase. Thus, this ABS was the most promising system for the separation of PDA_i_ while allowing the recovery and reuse of laccase. In addition, the colour obtained in the top phase after 1 h of reaction confirmed that the dopamine polymerization was successfully catalysed by laccase, Figure 3B.

From an integrated and sustainable process perspective, the dopamine partition is also important to better comprehend the composition of the phases and their reuse. Therefore, *EE*_*dopamine*_ % was determined and the results are depicted in Figure 3A. Dopamine preferentially migrated to the bottom phase for all ABS investigated, with an *EE*_*dopamine*_ for the bottom phase from 59.8 to 74.4%. Since dopamine is more hydrophilic than PDA_i_, this behaviour is expected due to the hydrophilic character of the bottom phase (You et al., 2017; Zhang et al., 2018). These results are promising, both dopamine and laccase migrate for the same phase, thus, allowing the recovery and reuse of the dopamine, that is not consumed by the enzymatic reaction, for posterior cycles of reaction.

Based on the obtained results, the ABS constituted by PPG 400 + K_2_HPO_4_ allowed a remarkable *EE*_*Laccase*_ of 100% for the bottom phase and PDA_i_ to the opposite top phase. Thus, this ABS was selected for further studies of enzyme reuse.

#### 3.2.2 Effect of dopamine concentration on the laccase-catalysed PDA_i_ using ABS

The polymerization of dopamine in the selected ABS was evaluated. Using the conditions previously selected, the reaction was carried out in a system composed of PPG 400 + K_2_HPO_4_/KH_2_PO_4_ at 30°C, pH 5.5 and initial dopamine concentration of 2.00 g ml^−1^. However, under these conditions, the formation of a precipitate was observed (Supplementary Figure S5, Supplementary Material), which did not allow to quantify the PDA_i_, the aim of this work. Thus, all the enzymatic reaction performed in ABS was carried out using the original pH of the system (pH 8.8).

**FIGURE 4:**
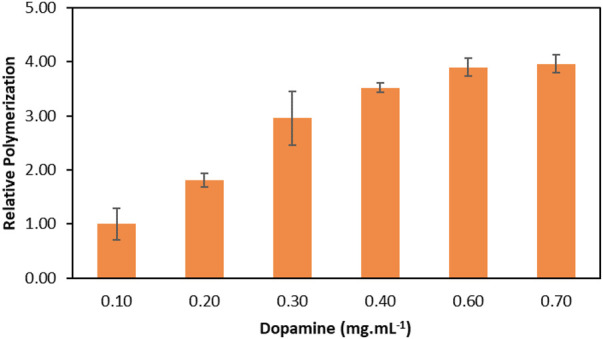
Effect of dopamine concentration in the relative enzymatic dopamine polymerization in relation to the conventional reaction (without laccase) in PPG 400 + K_2_HPO_4_ ABS after 1 h reaction at 30°C.

The optimisation of the laccase-catalysed dopamine polymerization was carried out in the selected ABS composed of PPG 400 + K_2_HPO_4_ by determining the ideal initial dopamine concentration in the ABS. The optimisation was evaluated based on the relative polymerization increase using different concentrations of dopamine, from 0.10 to 0.70 mg ml^−1^ after 1 h of reaction. Figure 4 depicts the relative polymerization fold of dopamine with laccase in the ABS, in relation to conventional reaction at the same conditions without laccase (considered as the control, 1-fold). The respective detailed data are reported in the Supplementary Material (Supplementary Table S7).

The results depicted in Figure 4 show an increase in the relative dopamine polymerization from 1.0 to 3.9-fold when dopamine concentration increases from 0.10 mg ml^−1^ up to 0.60 mg ml^−1^, respectively. Above this value, the relative dopamine polymerization remains constant, following a similar trend to that previously observed in Figure 2, which indicates enzyme saturation. Also, it is possible to conclude that all reactions with dopamine above 0.1 mg ml^−1^ lead to an outstanding polymerization increase in comparison with the conventional polymerization method performed without laccase, again demonstrating the high efficiency of the laccase-catalysed reaction.

Comparing to the performance of laccase in aqueous solution, a decrease in optimum dopamine concentration is observed. To explain this behaviour, the enzymatic activity of laccase was evaluated in the bottom phase of the ABS composed of PPG 400 + K_2_HPO_4_. Compared to the control (laccase in water at the same conditions), the laccase maintains 80% of the enzymatic activity in the K_2_HPO_4_ rich-phase. The decrease in laccase activity in the bottom phase can explain the difference in the relative polymerization between the results in the ABS and in aqueous solution. However, despite some loss in enzymatic activity in the ABS, this technique allows the recovery and reuse of the enzyme with good performance.

Since above 0.60 mg ml^−1^ the polymerization remains constant, this concentration was chosen for further assays. The choice of the initial concentration of dopamine is important to make the process profitable, making it more sustainable and economical, and avoiding the unnecessary use of the substrate.

### 3.3 ABS as liquid support for laccase recovery and reuse

**FIGURE 5:**
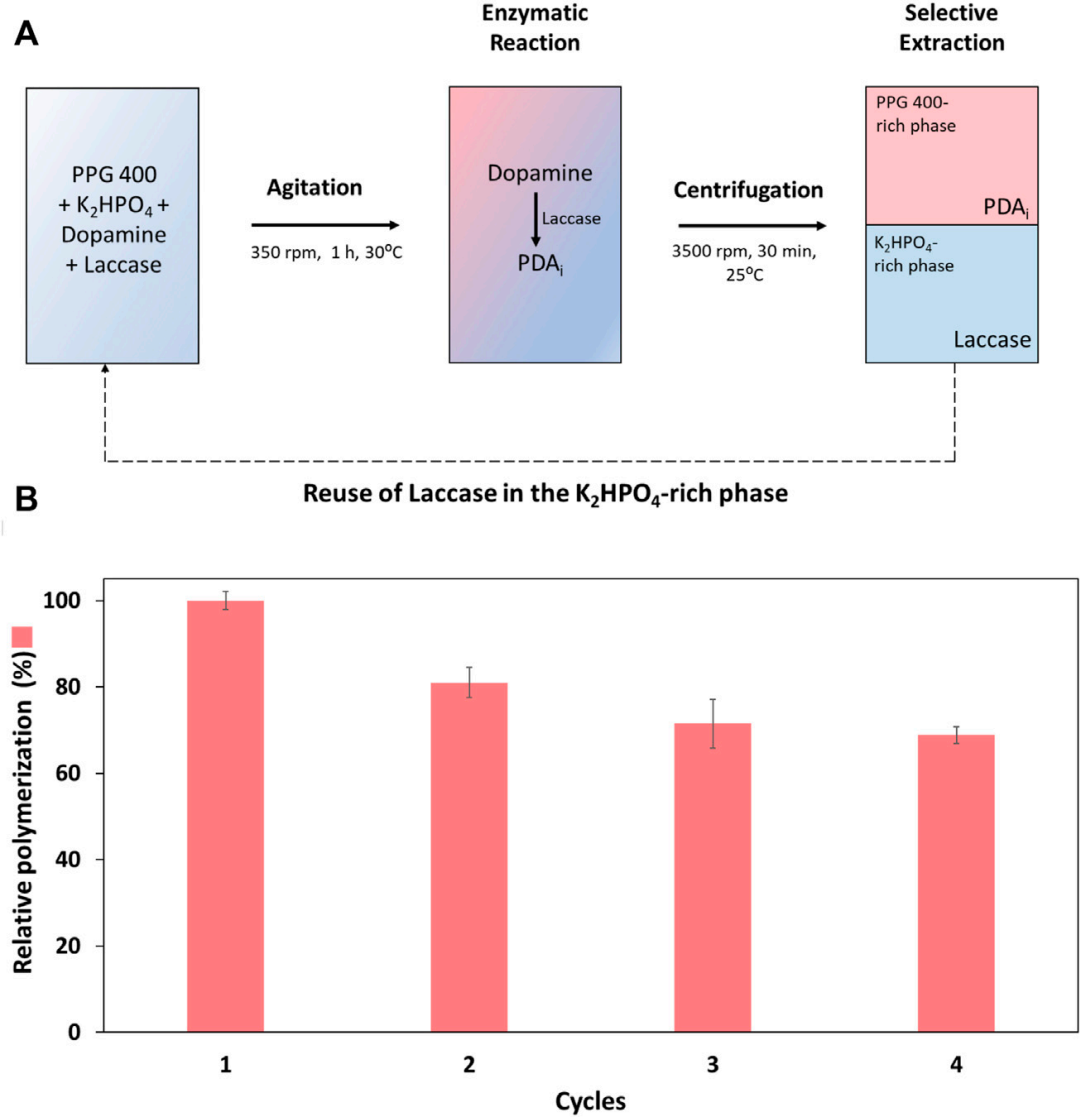
**(A)** Scheme of the integrated reaction—extraction process developed by applying ABS consisting of PPG 400 and K_2_HPO_4_, 30°C, including the recycling of the laccase and the K_2_HPO_4_-rich phase. **(B)** Relative dopamine polymerization (%) in four reaction cycles of the polymerization reaction (in relation to the first cycle).

After selecting the optimum dopamine concentration (0.60 mg ml^−1^), an integrated platform for laccase reuse and PDA_i_ recovery was developed using the ABS composed of PPG 400 + K_2_HPO_4_. The polymerization reaction was carried out in the ABS for four consecutive cycles. A scheme of the integrated reaction—extraction platform developed, including the recycling of the laccase and dopamine and the K_2_HPO_4_-rich phase is illustrated in Figure 5A. The enzymatic reaction in the ABS was carried out with continuous stirring for 1 h (heterogeneous medium). Then, the ABS was centrifuged, and the phases recovered. The relative polymerization, in relation to the first cycle, was determined, in Figure 5B [Detailed data are reported in the Supplementary Material (Supplementary Table S8)].

The enzymatic cycles were evaluated through the polymerization of dopamine into PDA_i_ (relative polymerization), Figure 5B. Overall, the recovery and reuse of laccase were possible for at least four reaction cycles, as can be confirmed by the obtained values of the relative polymerization (%) (Figure 5B). Despite some decrease in relative polymerization from the first to the second cycle, a remarkable relative polymerization of ∼70% in the last cycle was achieved. The decrease in the polymerization may be caused by the physical forces used, such as constant stirring and centrifugation throughout the four cycles of reaction, leading to enzymatic activity loss or by laccase inhibition caused by PDA_i_. Similar behaviour is reported in the literature, where intermediates of certain laccase reactions (e.g., lignin degradation) act as laccase inhibitors decreasing the enzyme activity (Pamidipati and Ahmed, 2020). To understand if a decrease in the enzymatic activity occurred, laccase activity was measured with ABTS substrate at the end of each cycle. A high decrease in laccase activity was obtained compared to the slight decrease in the relative polymerization (relative polymerization yield ∼70%, cycle 4). Thus, the influence of soluble PDA intermediates (PDA_i_ produced by the traditional method) in the laccase activity tests with ABTS was evaluated and compared to the control (activity of laccase in the presence of the buffer used in the production of PDA_i,_ considered 100%). The results presented in Supplementary Table S9 revealed a high influence of these compounds in the ABTS quantification method, the enzyme activity relative to the control is only 8.2%. Since soluble PDA_i_ are also laccase substrates for the formation of solid PDA, more than one substrate is competing for the active site of laccase during the measurement with ABTS and consequently contributing for the decrease in the values of enzyme activity during the cycles. In addition, the influence of dopamine in the ABTS method was also evaluated and as expected once dopamine is also laccase substrate, the enzyme activity relative to the control was 6% (Supplementary Table S9).

*EE*_*Laccase*_ % and *EE*_*PDA*_ % were also determined for all polymerization cycles (Supplementary Material, Supplementary Table S10). An *EE*_*Laccase*_ of 100% was obtained to the bottom phase throughout the four reaction cycles. Regarding *EE*_*PDA*_ %, 75.1% of PDA_i_ migrated to the top phase in the first reaction cycle, but it should be noted that some selectivity was lost at the end of the fourth cycle with an *EE*_*PDA*_ of 48.0%. However, in a continuous process of recycling, the PDA_i_ that migrated to the bottom phase will be returned along with the reused phase, before starting a new biocatalytic cycle, making the loss of PDA_i_ to the bottom phase not relevant.

According to the literature, only one work can be found on polymerization studies using laccase and ABS. This work studied the oligomerization of rutin in ABS composed of PEG 600 and [Ch][DHP] (Muñiz-Mouro et al., 2021). A total of three reaction cycles were achieved, with an oligomerization yield of 89% in the last one. It also verified a preferential migration of the laccase to the opposite phase of the product throughout the cycles (*EE*_*Laccase*_ % of ∼94% to the IL-rich phase). The *EE* of oligorutin was 67% and it was similar to that obtained in this study for PDA_i_ in the first cycle (∼75%).

The outcomes from successive cycles, considering good relative dopamine polymerization values, demonstrate the robustness of the reaction-extraction process proposed with ABS for integrated biocatalytic processes.

## 4 Conclusion

Laccase improves the dopamine polymerization reaction, achieving high polymerization rates when compared with the conventional polymerization method. The results show that PPG 400 + K_2_HPO_4_ ABS enable enzymatic processes while simultaneously extracting the products to the opposite phase. This selectivity demonstrates that it is possible to reuse the enzyme in a liquid support matching the ABS phase by allowing the extraction of the products from the enzyme. Therefore, ABS was here demonstrated as an efficient, and integrated reaction—separation platform to carry out the biocatalytic polymerization reactions, further allowing the reuse of the enzyme without compromising high polymerization rates and performance. For four consecutive reaction cycles, the phase containing the confined laccase was reused, with the final cycle having a relative polymerization of 68.9%. To sum up, it was found that the PPG 400 + K_2_HPO_4_ ABS is an efficient system since it allows the separation of PDA_i_ and laccase to opposite phases and the confinement and further reuse of laccase, thus, being an adequate liquid support for biocatalytic reactions and processes integration.

## Data Availability

The original contributions presented in the study are included in the article/Supplementary Material, further inquiries can be directed to the corresponding author.
