# Supplementary material for: New liquid supports in the development of integrated platforms for the reuse of oxidative enzymes and polydopamine production
Source: Front Bioeng Biotechnol. 2022 Nov 28;10:1037322. doi: 10.3389/fbioe.2022.1037322 (PMC9742376; doi:10.3389/fbioe.2022.1037322)
Supplement: Supplementary file 1 [file DataSheet1.pdf]

Supporting Information

New Liquid Supports in the Development of Integrated Platforms for the reuse of Oxidative Enzymes and Polydopamine Production

Flávia F. Magalhães[1†], Ana F. Pereira[1†], Mara G. Freire[1] and Ana P. M. Tavares[1*]

[1]CICECO-Aveiro Institute of Materials, Department of Chemistry, University of Aveiro, 3810-193, Aveiro, Portugal

*** Correspondence:**
Corresponding Author
aptavares@ua.pt

†These authors have contributed equally to this work and share first authorship

A)

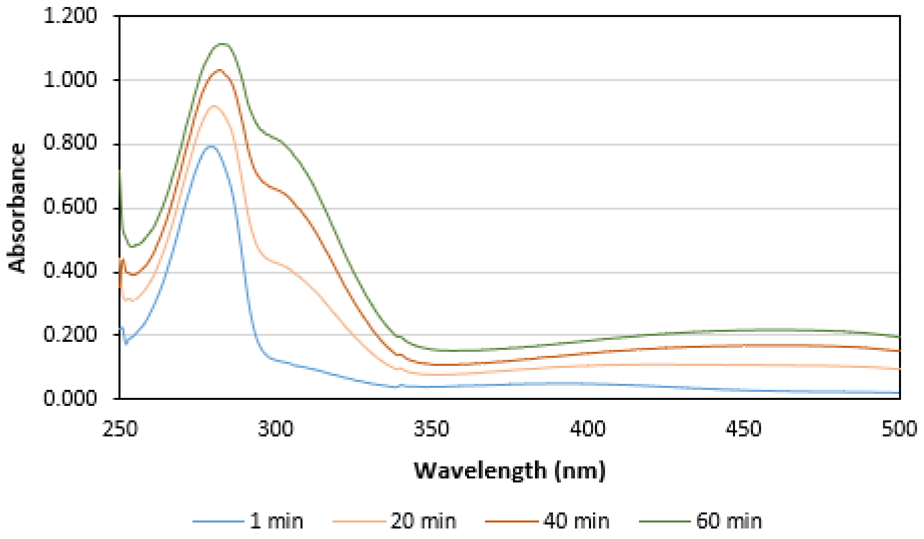

B)

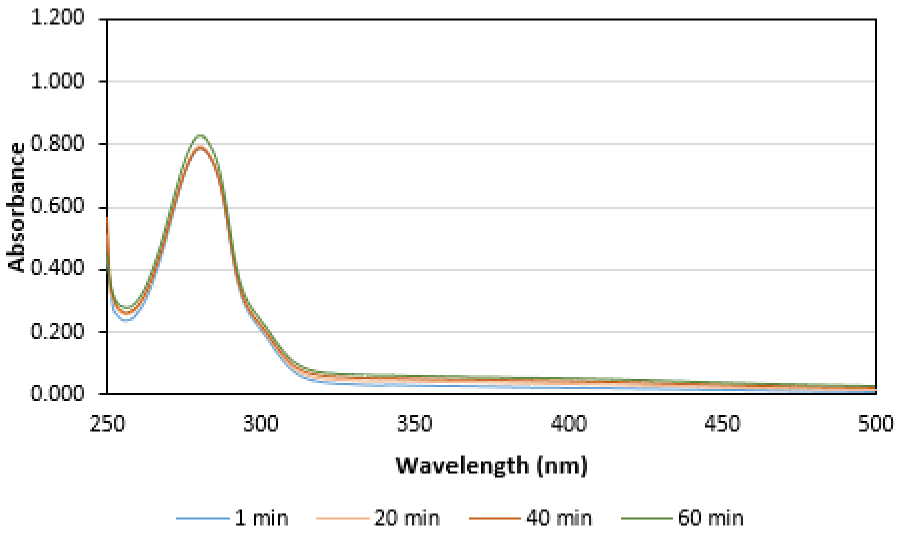

Figure S1. UV-Vis absorbance spectra of: **A)** enzymatic polymerization of dopamine, pH 5.5; **B)** conventional polymerization of dopamine, pH 8.5.

Regarding the enzymatic polymerization (Figure S1 A), the obtained spectra show a gradual increase in the absorbance intensity over all the reaction time, thus confirming the PDA formation

According to the literature, the characteristic absorption peak at 305 nm is assigned to dopamine semiquinone, an intermediate species whose subsequent oxidation originates dopaminequinone (Salomäki et al. 2018) (Figure S2).

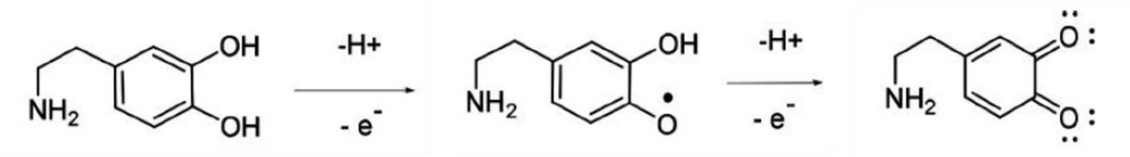

Figure S2. Initial pathway of dopamine oxidation with formation of dopaminequinone. Adapted from Salomäki et al. (Salomäki et al. 2018).

The absorbance intensity of this specie gradually increased over the time in the laccase catalysed reaction, whereas in the non-enzymatic reaction the peak at 305 nm remains practically unaltered. This can be explained because in the chemical oxidation of dopamine to the semiquinone form is slow, since this reaction is carried out under the O_2, a weak oxidizing agent, and also thermodynamically unfavourable for the whole pH range(Wu et al. 2020; Salomäki et al. 2018). In contrast, in the enzymatic reaction, a better polymerization performance was due to the oxidative capacity of the enzyme, which is related with the redox properties of the copper ions present in the active site of the enzyme allowing the intramolecular electron transfer from substrate oxidation from T1 copper centre to the T2/T3 redox trinuclear centre(Ivnitski and Atanassov 2007; Scheiblbrandner et al. 2017). According to this mechanism, the irreversible chemical oxidation of dopamine into dopaminequinone under the laccase-catalysed reaction occurs alongside the four-electron reduction of the O_2, the final electron acceptor which is finally reduced to two water molecules(Xiang et al. 2007) (Figure S3).

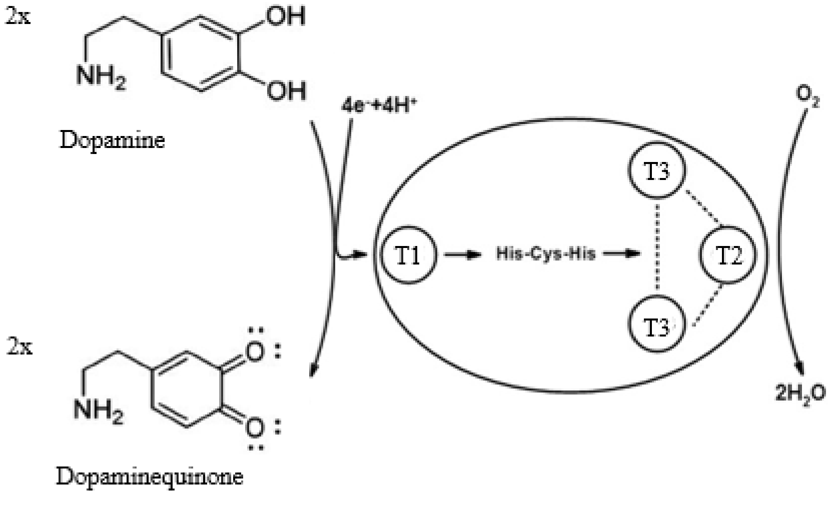

Figure S3. Simplified mechanism reaction of dopamine oxidation into dopaminequinone by laccase. Adapted by Wang *et al.*(Wang et al. 2015).

The choice of the absorption intensity at 480 nm is due to the characteristic absorption peak of dopaminechrome, another product from the oxidation of dopamine (Han, Tang, and Jin 2018; Salomäki et al. 2019, 2018), that undergoes rearrangement reactions and could polymerize to form PDA (Wei et al. 2010; Salomäki et al. 2018). This compound results from the intramolecular cyclization of the dopaminequinone to leucodopaminechrome, which in turn oxidizes to dopaminechrome(Salomäki et al. 2018; Wu et al. 2020). Thus, monitoring the variation of the absorption intensity at 480 nm of dopaminechrome is another trustworthy way to provide evidence of PDA formation. Several studies focus on the identification of the functional groups of this biopolymer as well as on the species involved in PDA formation through spectroscopy techniques. However, it should be noted that the exact structure of the resultant PDA is not yet fully known(Salomäki et al. 2018; Wu et al. 2020). Nevertheless, is it possible to calculate the molecular weight of the mentioned PDA intermediates: dopaminequinone, whose molecular formula is $C_8H_9NO_2$, has a molecular weight of 151.16 $g.mol^{-1}$, and dopaminechrome, with the molecular formula $C_9H_7NO_4$, has a molecular weight 193.16 $g.mol^{-1}$.

According to the spectrum of the enzyme catalysed reaction (Figure S1 A), a gradual increase in the absorbance intensity at 480 nm was obtained, whilst for the non-enzymatic case, there is a slight increase (Figure S1 B), as expected.

Considering the previous results obtained, the absorbance intensities at 305 and 480 nm as function of the time for enzymatic and non-enzymatic polymerization of PDA are represented in Figure S4.

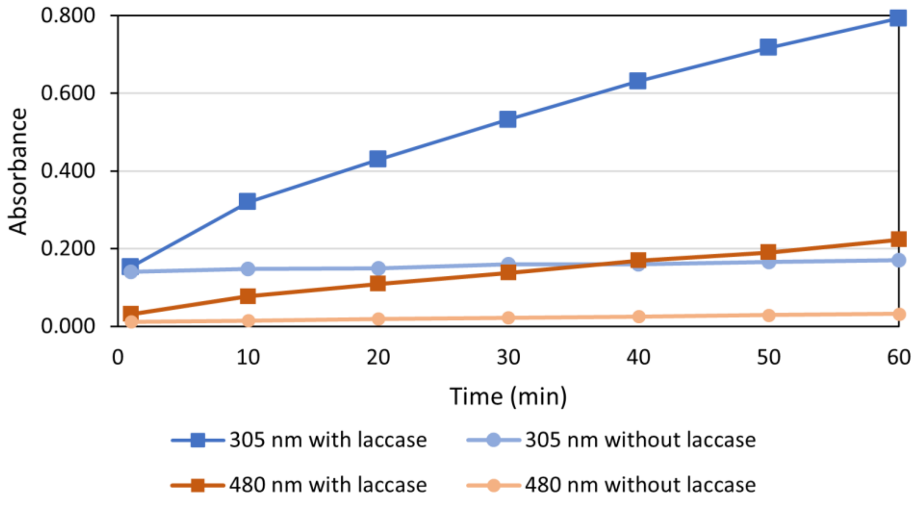

Figure S4. Absorbance values at 305 and 480 nm of dopamine polymerization catalysed with and without laccase.

As shown in Figure S4, there is a fast increase in the absorption intensities at 305 and 480 nm of the enzymatic polymerization during the entire reaction time, although this increase is more remarkable at 305 nm. In contrast, in the non-enzymatic case there is a slow and slight increase for both absorbance intensities. The dopamine polymerization reaction was improved in the presence of laccase since for the same reaction time the polymerization was significantly superior.

Optimization of enzymatic polymerization of dopamine

Table S1. Relative dopamine polymerization (fold) for the enzymatic polymerization, in relation to the non-enzymatic reaction, at different pH values and 30 °C and 2.00 mg.mL^{-1} of dopamine.

**pH**	**Relative Polymerization (fold)**
4.5	4.60 ± 0.18
5.5	7.20 ± 0.58
6.5	4.80 ± 0.03
7.5	2.29 ± 0.37
8.5	1.00 ± 0.13

Table S2. Relative dopamine polymerization (fold) for the enzymatic polymerization, in relation to the non-enzymatic reaction, at different temperatures and pH 5.5 and 2.00 mg.mL^{-1} of dopamine.

**Temperature (ºC)**	**Relative Polymerization (fold)**
20	5.52 ± 0.36
25	5.84 ±0.65
30	7.20 ± 0.58
35	6.32 ± 0.50
40	5.84 ± 0.23

Table S3. Relative Dopamine Polymerization (fold) for the enzymatic polymerization, in relation to the non-enzymatic reaction, at different dopamine concentrations, pH 5.5 and 30 °C.

**Dopamine concentration ($mg.mL^{-1}$)**	**Relative Polymerization (fold)**
0.25	3.22 ± 0.12
0.50	5.81 ± 0.08
1.0	6.47 ± 0.33
1.5	6.88 ± 0.06
2.0	7.42 ± 0.21
2.5	7.24 ± 0.28
3.0	7.22 ± 0.24

Extraction efficiency of laccase, PDA and dopamine

Table S4. Extraction efficiency of PDA (EE_{PDA} %) to the top phase.

**ABS**	**EE_{PDA} (%)**
PPG 400 + [Ch][DHP]	32.2 ± 2.6
PPG 400 + [Ch][DHC]	31.1 ± 4.0
PPG 400 + [Ch][Acet]	53.8 ± 2.6
PPG 400 + K_2HPO_4	77.8 ± 1.0
PPG 400 + PEG 400	17.2 ± 1.8

Table S5. Extraction efficiency of laccase ($EE_{Laccase}$ %) to the bottom phase.

**ABS**	**$EE_{Laccase}$ (%)**
PPG 400 + [Ch][DHP]	100.0 ± 0.0
PPG 400 + [Ch][DHC]	100.0 ± 0.0
PPG 400 + [Ch][Acet]	100.0 ± 0.0
PPG 400 + K_2HPO_4	100.0 ± 0.0
PPG 400 + PEG 400	100.0 ± 0.0

Table S6. Extraction efficiency of dopamine ($EE_{Dopamine}$ %) to the top and bottom phase.

**ABS**	$EE_{Dopamine}$ **(%) Top-phase**	$EE_{Dopamine}$ **(%) Bottom-phase**
PPG 400 + [Ch][DHP]	25.6 ± 3.2	74.4 ± 3.2
PPG 400 + [Ch][DHC]	30.5 ± 2.5	69.5 ± 2.5
PPG 400 + [Ch][Acet]	30.3 ± 1.3	69.7 ± 1.3
PPG 400 + K_2HPO_4	40.2 ± 1.3	59.8 ± 1.3
PPG 400 + PEG 400	31.3 ± 1.7	68.7 ± 1.7

Table S7. Relative dopamine polymerization (fold) for the enzymatic polymerization in relation to the non-enzymatic reaction, at different dopamine concentrations using ABS as the rection medium at 30 ºC.

**Dopamine concentration (mg mL^{-1})**	**Relative Polymerization (fold)**
0.10	1.00 ± 0.29
0.20	1.81 ± 0.13
0.30	2.96 ± 0.49
0.40	3.52 ± 0.08
0.60	3.90 ± 0.17
0.70	3.96 ± 0.17

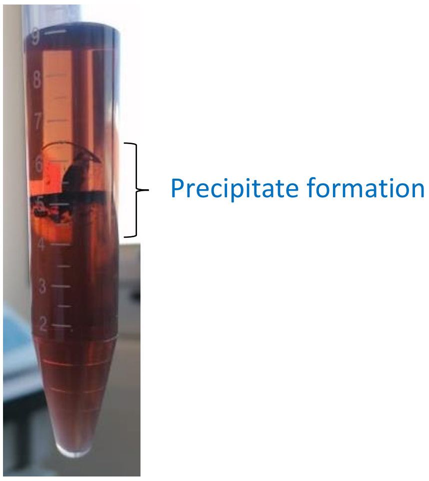

Figure S5. ABS composed of PPG 400 + potassium phosphate buffer solution composed of K_2HPO_4 and KH_2PO_4 salts at pH = 5.5; It is possible to observe the ocurrence of a precipitate in the interphase.

Table S8. Relative polymerization (%) of dopamine in PPG 400-rich phase during 4 reaction cycles of polymerization reaction.

**Cycle**	**Relative Polymerization (%)**
1st	100.0 ± 2.1
2nd	81.0 ± 3.5
3rd	71.5 ± 5.7
4th	68.9 ± 2.0

Table S9. Relative laccase activity (%) in the presence of dopamine and PDA_i in relation to the control (laccase solution).

	**Dopamine concentration (mg mL^{-1})**			**In the presence of PDA_i**
	0.1	0.5	1.0	
Relative Laccase Activity (%)	6.0 ± 1.0	0.7 ± 0.0	0.7 ± 0.3	8.2 ± 0.9

Table S10. $EE_{Laccase}$ *(%)* to the bottom phase and EE_{PDA} *%* to the top phase in 4 reaction cycles of polymerization reaction.

Cycle	$EE_{Laccase}$ (%)	EE_{PDA} (%)
1st	100.0 ± 0.0	75.1 ± 3.5
2nd	100.0 ± 0.0	64.0 ± 2.2
3rd	100.0 ± 0.0	52.1 ± 2.1
4th	100.0 ± 0.0	48.0 ± 0.2

References

Han, Xuwen, Feng Tang, and Zhaoxia Jin. 2018. “Free-Standing Polydopamine Films Generated in the Presence of Different Metallic Ions: The Comparison of Reaction Process and Film Properties.” *RSC Advances* 8 (33): 18347–54. https://doi.org/10.1039/c8ra02930j.

Ivnitski, Dmitri, and Plamen Atanassov. 2007. “Electrochemical Studies of Intramolecular Electron Transfer in Laccase from Trametes Versicolor.” *Electroanalysis* 19 (22): 2307–13. https://doi.org/10.1002/elan.200703983.

Salomäki, Mikko, Lauri Marttila, Henri Kivelä, Tuomo Ouvinen, and Jukka Lukkari. 2018. “Effects of PH and Oxidants on the First Steps of Polydopamine Formation: A Thermodynamic Approach.” *The Journal of Physical Chemistry B* 122 (24): 6314–27. https://doi.org/10.1021/acs.jpcb.8b02304.

Salomäki, Mikko, Tuomo Ouvinen, Lauri Marttila, Henri Kivelä, Jarkko Leiro, Ermei Mäkilä, and Jukka Lukkari. 2019. “Polydopamine Nanoparticles Prepared Using Redox-Active Transition Metals.” *Journal of Physical Chemistry B* 123 (11): 2513–24. https://doi.org/10.1021/acs.jpcb.8b11994.

Scheiblbrandner, Stefan, Erik Breslmayr, Florian Csarman, Regina Paukner, Johannes Führer, Peter L. Herzog, Sergey V. Shleev, et al. 2017. “Evolving Stability and PH-Dependent Activity of the High Redox Potential Botrytis Aclada Laccase for Enzymatic Fuel Cells.” *Scientific Reports* 7 (1): 1–13. https://doi.org/10.1038/s41598-017-13734-0.

Wang, Jinhui, Juanjuan Feng, Weitao Jia, Sandra Chang, Shizhong Li, and Yinxin Li. 2015. “Lignin Engineering through Laccase Modification: A Promising Field for Energy Plant Improvement.” *Biotechnology for Biofuels* 8 (1): 1–12. https://doi.org/10.1186/s13068-015-0331-y.

Wei, Qiang, Fulong Zhang, Jie Li, Beijia Li, and Changsheng Zhao. 2010. “Oxidant-Induced Dopamine Polymerization for Multifunctional Coatings.” *Polymer Chemistry* 1 (9): 1430. https://doi.org/10.1039/c0py00215a.

Wu, Meng, Tao Wang, Lenka Müller, and Frank A. Müller. 2020. “Adjustable Synthesis of Polydopamine Nanospheres and Their Nucleation and Growth.” *Colloids and Surfaces A: Physicochemical and Engineering Aspects* 603 (June): 125196. https://doi.org/10.1016/j.colsurfa.2020.125196.

Xiang, Ling, Yuqing Lin, Ping Yu, Lei Su, and Lanqun Mao. 2007. “Laccase-Catalyzed Oxidation and Intramolecular Cyclization of Dopamine: A New Method for Selective Determination of Dopamine with Laccase/Carbon Nanotube-Based Electrochemical Biosensors.” *Electrochimica Acta* 52 (12): 4144–52. https://doi.org/10.1016/j.electacta.2006.11.040.
